# Magnetic resonance evaluation of three-dimensional liver fat fraction by hepatitis C status and associations with inflammatory cytokines

**DOI:** 10.1371/journal.pone.0327668

**Published:** 2025-07-23

**Authors:** Jessie Torgersen, Craig W. Newcomb, Dean M. Carbonari, Shanae M. Smith, Katherine L. Brecker, Chamith S. Rajapakse, Brandon C. Jones, Christiana Cottrell, Rasleen Grewal, Jennifer C. Price, Joshua F. Baker, Jay R. Kostman, Stacey Trooskin, Rebecca A. Hubbard, Babette S. Zemel, Mary B. Leonard, Vincent Lo Re III

**Affiliations:** 1 Department of Medicine, Perelman School of Medicine, University of Pennsylvania, Philadelphia, Pennsylvania, United States of America; 2 Department of Biostatistics, Epidemiology, and Informatics, Center for Clinical Epidemiology and Biostatistics, Center for Real-world Effectiveness and Safety of Therapeutics, Perelman School of Medicine, University of Pennsylvania, Philadelphia, Pennsylvania, United States of America; 3 Department of Radiology, Perelman School of Medicine, University of Pennsylvania, Philadelphia, Pennsylvania, United States of America; 4 Department of Orthopedic Surgery, Perelman School of Medicine, University of Pennsylvania, Philadelphia, Pennsylvania, United States of America; 5 Department of Bioengineering, University of Pennsylvania, Philadelphia, Pennsylvania, United States of America; 6 Department of Medicine, University of California, San Francisco School of Medicine, San Francisco, California, United States of America; 7 Philadelphia FIGHT, Philadelphia, Pennsylvania, United States of America; 8 Mazzoni Center, Philadelphia, Pennsylvania, United States of America; 9 Department of Biostatistics, Brown University School of Public Health, Providence, Rhode Island, United States of America; 10 Department of Pediatrics, Children’s Hospital of Philadelphia, Philadelphia, Pennsylvania, United States of America; 11 Department of Pediatrics, Stanford University School of Medicine, Palo Alto, California, United States of America; Johns Hopkins University School of Medicine, UNITED STATES OF AMERICA

## Abstract

**Background:**

Chronic hepatitis C virus (HCV) infection may influence cytokine and insulin-like growth factor (IGF-1) levels, which could contribute to increased hepatic steatosis. We utilized MRI to compare three-dimensional volumetric liver fat fraction by chronic HCV status and evaluated associations between liver fat fraction and inflammatory cytokines and IGF-1.

**Methods:**

Participants with untreated, non-genotype 3 chronic HCV and participants without HCV were enrolled between 2019−2022 and underwent MRI to quantify three-dimensional volumetric liver fat fraction. Interleukin (IL)-6, IL-18, tumor necrosis factor (TNF)-α, and IGF-1 were also measured. Multivariable linear regression was used to determine associations between liver fat fraction, chronic HCV, and cytokine and IGF-1 levels.

**Results:**

Among 54 participants with HCV and 54 without HCV, median volumetric liver fat fraction was 12.4% (IQR: 9.3, 18.0%) and 10.9% (IQR: 8.7, 13.3%), respectively. After adjustment for age, sex, and body mass index, mean liver fat fraction was 2.28% (95% CI: 0.55, 4.02%) higher in participants with HCV. HCV was associated with higher mean log TNF-α (0.11 [95% CI: 0.06, 0.16]) and IL-18 (0.14 [95% CI: 0.05, 0.24]), but lower mean log IGF-1 (−0.18 [95% CI: −0.26, −0.11]) when compared to those without HCV. IL-6, IL-18, TNF-α, and IGF-1 were not associated with liver fat fraction.

**Conclusion:**

Chronic HCV is associated with higher volumetric liver fat fraction by MRI. TNF-α and IL-18 levels were higher with chronic HCV but were not associated with liver fat fraction. Further research is needed to identify alternative mechanisms that potentiate liver fat deposition in chronic HCV.

## Introduction

Hepatic steatosis, defined by >5% hepatocyte lipid accumulation, is common among people living with chronic hepatitis C virus (HCV) infection and can contribute to progression of liver disease [1]. Cross-sectional studies employing histopathologic assessment of liver tissue estimate that between 40–86% of people with chronic HCV also have hepatic steatosis [2]. Although these studies relied on liver biopsy for hepatic steatosis quantification, they may be subject to sampling bias, since percutaneous liver biopsy only samples 1/50,000^th^ of the liver [3]. Moreover, such studies may be subject to indication bias as people with more severe disease have been more likely to undergo liver biopsy [4]. Image-based assessments of hepatic steatosis have aimed to overcome this limitation of liver biopsy. Notably, magnetic resonance imaging (MRI) proton density fat fraction has been identified as highly accurate in the evaluation of steatotic liver disease [5]. Yet, the two-dimensional nature of standard MRI approaches may incompletely evaluate liver fat. Since hepatic steatosis may not be homogeneous in distribution [6], three-dimensional volumetric assessment may provide a more comprehensive determination of liver fat throughout the hepatic parenchyma.

Chronic HCV can lead to accumulation of liver fat through virally-mediated alterations in lipid metabolism [7,8]. Down-regulation of fatty acid breakdown and export of cholesterol occurs due to viral interference prioritizing virion maturation and secretion, resulting in an accumulation of hepatic lipids [7]. Additionally, increased oxidative stress due to chronic HCV infection contributes to increased lipid peroxidation, leading to an imbalance in lipid homeostasis, promoting intracellular accumulation of lipid droplets [8]. However, it remains unclear if HCV directly contributes to the development of liver fat accumulation since prior studies have demonstrated lower prevalence [9,10], no difference [11,12], and higher prevalence [13,14] of hepatic steatosis in people with HCV infection compared to those without HCV.

Chronic HCV infection might further potentiate hepatic steatosis through systemic inflammatory cytokine activation and the development of hepatic fibrosis. Cytokines, particularly interleukin (IL)-6, IL-18, and tumor necrosis factor (TNF)-α, could promote intra-hepatic lipid peroxidation and peripheral lipolysis, contributing to hepatic steatosis [15]. HCV-induced hepatic fibrosis could also contribute to hepatic and systemic metabolic dysregulation through impaired production of insulin-like growth factor (IGF)-1 in the liver [16]. Together with traditional risk factors for steatosis, particularly visceral adiposity, hyperlipidemia, insulin resistance, and alcohol use [17–27], these factors may underlie mechanisms contributing to the development of HCV-associated hepatic steatosis.

We utilized MRI to compare three-dimensional volumetric liver fat fraction among participants with and without chronic HCV infection. To extend understanding of the determinants of hepatic steatosis, we next evaluated associations between liver fat fraction and inflammatory cytokines and IGF-1. Understanding associations between HCV infection, liver fat fraction, and the role of inflammation could shed light on potential mechanisms for the development of hepatic steatosis in these patients.

## Materials and methods

### Study design and setting

We conducted a cross-sectional study of patients with and without chronic HCV infection who were recruited from infectious diseases, hepatology, and family/general medicine practices at the Hospital of the University of Pennsylvania, Penn Presbyterian Medical Center, and the Philadelphia FIGHT, all located in Philadelphia, Pennsylvania. These practices provide HCV and general medical care to people with and without HCV.

Patients scheduled for routine clinical visits at participating clinical locations were pre-screened for HCV status and approached by study personnel after discussion with their healthcare provider. All patients were initially screened for eligibility via self-reported responses. Potentially eligible patients who provided informed consent had their eligibility verified by medical record review. To ensure balance in participant demographics between the groups, participants without HCV infection were sampled within strata to balance the distribution of sex, race (Black versus not Black), and age category (18–39, 40–59, ≥ 60 years) relative to participants with chronic HCV. Participants were recruited between January 1, 2019 and July 31, 2022. The study was approved by the Institutional Review Board (IRB) of the University of Pennsylvania, which served as the IRB of Record. The Philadelphia FIGHT IRB relied on the University of Pennsylvania IRB through a reliance agreement. Written informed consent was obtained from all participants.

### Study participants

Participants with chronic HCV infection were eligible for inclusion if, at enrollment, they had: 1) ≥18 years of age; 2) detectable HCV RNA, 3) hepatic fibrosis staging within the previous 6 months, and 4) provided informed consent. Participants with chronic HCV who had HIV coinfection were eligible if they had an HIV RNA < 200 copies/mL on a stable antiretroviral therapy regimen for ≥4 weeks prior to enrollment.

Participants without HCV infection were eligible for inclusion if, at enrollment, they were: 1) ≥18 years of age; 2) HIV negative, and 3) able to provide informed consent. Participants without HCV infection were confirmed to be HCV and HIV antibody-negative by OraSure’s OraQuick® assay administered by study personnel at the study visit.

Participants were excluded if they had conditions that might affect body composition or liver fat, including: chronic kidney disease (estimated glomerular filtration rate <60 mL/min/1.73 m^2^) [28]; malignancy (excluding non-melanomatous skin cancer); diseases that promote malabsorption (i.e., celiac disease, small bowel resection surgery, chronic diarrhea); weight loss of >5% of body weight over the preceding three months); or another chronic liver disease (i.e., hepatitis B virus infection, hemochromatosis, α-1-antitrypsin, autoimmune hepatitis, primary sclerosing cholangitis, primary biliary cirrhosis, Wilson’s disease, or non-alcoholic fatty liver disease or steatohepatitis; see Participant Screening Questions in S1 File). We also excluded patients who were pregnant. Participants with chronic HCV were additionally excluded if they were: 1) infected with HCV genotype 3 (since this promotes hepatic steatosis [29,30]), or 2) previously received direct-acting antiviral-based HCV therapy (since treatment might affect steatosis [31]). We estimated that 50 participants per group would provide 80% power to detect a difference in liver fat fraction at the two-sided alpha = 0.05-level of at least 5.0% between the groups, which we considered clinically significant.

### Assessment of demographic, clinical, and anthropometric data

Data collected from participants included: date of birth; sex assigned at birth; race; ethnicity; current smoking status; recent alcohol consumption; recent drug use; history of injection drug use; and postmenopausal status (if applicable; defined as either: 1) absence of menstrual periods for >12 months in a previously menstruating individual >45 years of age while not on hormonal birth control, or 2) underwent hysterectomy or bilateral oophorectomy). Alcohol consumption was determined by the 10-question Alcohol Use Disorders Identification Test (AUDIT) [32], and drug use was determined by the 10-question Drug Abuse Screening Test (DAST-10) [33].

Diabetes mellitus (classified based on diagnosis, hemoglobin A1c ≥ 6.5%, or random glucose ≥200 mg/dL within 12 months prior to study visit) and most recent serum creatinine results recorded within 12 months prior to study visit were obtained from participants’ medical records. For participants with chronic HCV, we also collected earliest self-reported HCV diagnosis date, most recent HCV RNA, HCV genotype, and stage of liver fibrosis by vibration-controlled transient elastography or proprietary serologic fibrosis assay [34]. Low/minimal hepatic fibrosis was defined by METAVIR stages 0–2, and advanced hepatic fibrosis/cirrhosis was defined as METAVIR stage F3-F4 [34].

Body weight and height were measured in triplicate without shoes using a digital scale (Scaltronix; Skaneateles, NY) and stadiometer (Holtain; Crymych, UK), respectively, and the mean of each used for analysis. Body mass index (BMI) was calculated as weight in kilograms divided by height in meters squared.

### Measurement of hepatic and visceral adipose depots

A 3-Tesla whole-body MR imager (Siemens Prisma; Erlangen, Germany) was used for three-dimensional volumetric assessment of liver fat fraction and cross-sectional area of abdominal visceral and subcutaneous adipose tissue (see details in S2 File). For the liver fat fraction analysis, radiology research technicians segmented the liver boundary on all slices from the fourth echo image (S1 Fig). Results were confirmed via independent review by a radiology researcher with over two decades in body MRI research. The total liver volume was estimated by multiplying the number of voxels within the three-dimensional segmented volume-of-interest by the voxel size (2x2x5 mm). For computation of liver fat fraction, a morphological erosion of one voxel was first applied to the outer layer of the three-dimensional segmented liver volume-of-interest to mitigate any partial-volume effects along the liver boundary. The liver fat fraction was then computed as the voxel-wise median fat fraction (%) across the entire three-dimensional volume-of-interest.

### Laboratory data

Blood samples were collected for determination of serum total cholesterol, high-density lipoprotein, low-density lipoprotein, and triglyceride levels (Roche Cobas c311; Roche Diagnostics). Serum levels of IL-6, IL-18, and TNF-α were measured using an enzyme-linked immunosorbent assay (ProteinSimple Ella; Bio-Techne; Minneapolis, MN). IGF-1 was measured using an electrochemiluminescence analyzer (Roche Cobas e411; Roche Diagnostics).

### Statistical analysis

We first evaluated differences in liver fat fraction, significant hepatic steatosis (defined by liver fat fraction >5%) [35], and subcutaneous and visceral adipose tissue area by HCV status. We then used multivariable linear regression to determine the mean difference (95% confidence interval [CI]) in liver fat fraction between participants with and without chronic HCV infection, after adjustment for age (continuous), sex, and BMI (continuous). In these linear regression analyses, the null hypothesis was that there was no difference in liver fat fraction between groups (mean difference in liver fat fraction = 0 between the groups); statistically significant differences in liver fat fraction were declared if the 95% CI excluded zero [36]. To assess the robustness of the results, we repeated our analyses replacing BMI with visceral fat mass cross-sectional area by MRI.

We conducted three exploratory analyses. First, we stratified the HCV group by HIV status and determined the mean difference in liver fat fraction between participants with HCV/HIV coinfection and people without HIV or HCV. Second, we stratified the HCV group by liver fibrosis stage (low/minimal hepatic fibrosis versus advanced hepatic fibrosis/cirrhosis) and determined the mean difference in liver fat fraction compared to people without HIV or HCV. Since the correlations between BMI and abdominal subcutaneous or visceral adipose depots are unclear in advanced liver disease, a state in which sarcopenia can develop [37], we modeled the association between liver fibrosis stage and abdominal subcutaneous adipose tissue and visceral adipose tissue, independent of BMI. Third, among participants with chronic HCV, we evaluated the association between log HCV RNA and liver fat fraction. The linearity of continuous variables was confirmed by examination of scatterplots for residuals.

Next, we used multivariable linear regression to determine the mean difference (95% CI) in log IL-6, IL-18, TNF-α, and log IGF-1 levels between participants with and without HCV infection, after adjustment for age (continuous), sex, and BMI (continuous). We log transformed these measurements to improve linearity. We also examined whether hepatic fibrosis stage modified the results.

We then evaluated associations between log level of specified cytokine and IGF-1 and liver fat fraction. Using multivariable linear regression, we determined the mean difference (95% CI) in liver fat fraction per log level of IL-6, IL-18, TNF-α, and log IGF-1, after adjustment for age (continuous), sex, BMI (continuous), and HCV status.

Finally, to explore whether there was a relationship between chronic HCV and abdominal subcutaneous fat or visceral fat, we separately evaluated associations between chronic HCV and both abdominal subcutaneous adipose tissue and visceral adipose tissue cross-sectional area, after adjusting for age, sex, and BMI. We further explored the impact of hepatic fibrosis stage on these associations.

All analyses were completed using SAS v9.4 (SAS Institute Inc.).

## Results

### Participant characteristics

Among 519 patients screened for eligibility, 172 were potentially eligible and interested. After medical record review, 54 participants with untreated chronic HCV infection and 54 participants without HCV were enrolled (S2 Fig). The characteristics of the participants are summarized in Table 1. No differences were observed by HCV status for median age at enrollment, race, ethnicity, diagnosis of diabetes, or dyslipidemia. Median BMI and median AUDIT-C score were modestly higher in people without HCV; however, no statistically significant difference was observed. Current smoking status was more common among people with HCV. Among participants with chronic HCV, 12 (22%) were living with HIV.

**Table 1:** Characteristics of study participants who completed enrollment study visit, by chronic hepatitis C virus infection status.

Characteristic^a^	Participants with Chronic HCV (n = 54)	Participants Without HCV (n = 54)	*P*-Value
**Age, median (IQR), years**	55 (42, 62)	53 (36, 62)	0.70
**Male sex**	41 (75.9%)	32 (59.3%)	0.06
**Race**			
White	18 (33.3%)	18 (33.3%)	0.35
Black or African American	34 (63.0%)	31 (57.4%)	
Asian	0 (0%)	2 (3.7%)	
Other	2 (3.7%)	1 (1.9%)	
Multiracial	0 (0%)	2 (3.7%)	
**Hispanic ethnicity**	4 (7.4%)	4 (7.4%)	1.00
**Body mass index**			
Median (IQR)	27 (24, 30)	29 (25, 33)	0.06
Underweight (<18.50 kg/m^2^)	0 (0.0%)	0 (0.0%)	0.20
Normal (18.50–24.99 kg/m^2^)	17 (31.5%)	14 (25.9%)	
Overweight (25.00–29.99 kg/m^2^)	21 (38.9%)	15 (27.8%)	
Obesity (≥30.00 kg/m^2^)	16 (29.6%)	25 (46.3%)	
**Fat mass index, median (IQR), kg/m** ^2^	7.8 (5.6, 10.0)	9.3 (6.3, 12.3)	0.04
**Diabetes mellitus** ^b^	12 (22.2%)	10 (18.5%)	0.63
**Alcohol use in past year, AUDIT score**			
Median (IQR)	1 (0, 5)	2 (1, 4)	0.05
< 8 (low risk)	48 (88.9%)	49 (90.7%)	0.94
8–15 (risky/hazardous)	5 (9.3%)	4 (7.4%)	
16–19 (high-risk)	0 (0.0%)	0 (0.0%)	
≥ 20 (highest risk)	1 (1.9%)	1 (1.9%)	
**Drug use in past year, DAST-10 score**			
Median (IQR)	1 (0, 7)	0 (0, 1)	0.01
0 (none)	24 (44.4%)	38 (70.4%)	<.0001
1–2 (low)	8 (14.8%)	15 (27.8%)	
3–5 (moderate)	5 (9.3%)	1 (1.9%)	
6–8 (substantial)	11 (20.4%)	0 (0%)	
9–10 (severe)	6 (11.1%)	0 (0%)	
**Smoking pack years, median (IQR)** ^c^	8.75 (5.00, 12.00)	8.00 (2.60, 13.50)	0.75
**History of injection drug use**	30 (55.6%)	2 (3.7%)	<.0001
**Median serum creatinine (mg/dL, IQR)** ^d^	0.93 (0.81, 1.02)	0.96 (0.80, 1.07)	0.67
Missing	2 (3.7%)	16 (29.6%)	0.0003
**Median total cholesterol (mg/dL, IQR)**	161 (141, 186)	169 (147, 187)	0.40
Missing	6 (11.1%)	1 (1.9%)	0.05
**Median HDL (mg/dL, IQR)**	50 (41, 65)	52 (45, 61)	0.48
Missing	6 (11.1%)	1 (1.9%)	0.05
**Median LDL (mg/dL, IQR)**	97 (79, 114)	93 (75, 119)	0.37
Missing	6 (11.1%)	1 (1.9%)	0.05
**Median triglycerides (mg/dL, IQR)**	105 (79, 129)	93 (64, 155)	0.62
Missing	6 (11.1%)	1 (1.9%)	0.05
**Dyslipidemia** ^e^	20 (37.0%)	20 (37.0%)	1.00
Missing	6 (11.1%)	1 (1.9%)	0.05
**HIV infection**	12 (22.2%)	--	--
**Hepatic fibrosis stage** ^f^			
F0-F2	42 (77.8%)	--	--
F3-F4	11 (20.4%)	--	--
**Hepatitis C genotype**			
1	1 (1.9%)	--	--
1a	38 (70.4%)	--	--
1b	12 (22.2%)	--	--
2	1 (1.9%)	--	--
2b	2 (3.7%)	--	--
**Time since HCV diagnosis (years)** ^g^			
0–1 year	20 (37.0%)	--	--
2–4 years	12 (22.2%)		
5–10 years	6 (11.1%)	--	--
11–20 years	10 (18.5%)	--	--
> 20 years	6 (11.1%)	--	--

Abbreviations: AUDIT = Alcohol Use Disorders Identification Test; DAST = Drug Abuse Screen Test; HCV = hepatitis c virus; HDL = high-density lipoproteins; IQR = interquartile range; LDL = low-density lipoproteins

^a^Results reported as n (%) unless otherwise noted.

^b^Diabetes mellitus was defined as the presence of any of the following: diagnosis of diabetes ever in the medical record, or within the prior 12 months, either a) hemoglobin A1c ≥ 6.5%, or b) random glucose ≥200 mg/dL.

^c^For current smokers, smoking pack years was calculated as the average number of cigarettes smoked per day divided by 20, then multiplied by the number of years the person has smoked.

^d^Serum creatinine levels from the prior 12 months, if available, were collected from the medical record.

^e^Dyslipidemia was defined as triglycerides ≥150 mg/dL or HDL either <40 mg/dL if male or <50 mg/dL if female.

^f^METAVIR fibrosis stage approximated from vibration-controlled transient elastography or proprietary serologic fibrosis assay. One participant with chronic HCV had an invalid fibrosis assessment.

^g^Time since HCV diagnosis based on self-reported year of initial HCV diagnosis.

### Mean differences in liver fat fraction, by HCV status

Median liver fat fraction, as measured by MRI using a three-dimensional volumetric evaluation, was 12.4% (95% CI: 9.3, 18.0%) among participants with chronic HCV infection and 10.9% (8.7, 13.3%) among participants without HCV (Table 2). The prevalence of significant hepatic steatosis (i.e., > 5% fat fraction) was 100% among people with HCV and 100% among people without HCV, with maximum liver fat fraction less than 33% in both groups. After adjustment for age, sex, and BMI, participants with chronic HCV had 2.28% (95% CI: 0.55, 4.02%) higher mean liver fat fraction than participants without HCV (Table 3). Mean liver fat fraction remained higher for participants with chronic HCV when analyses were repeated replacing BMI with visceral fat cross-sectional area (Table 3).

**Table 2:** Liver and abdominal composition measurements by magnetic resonance imaging among participants with and without chronic hepatitis C virus infection.

Measurement^a^	Participants with Chronic HCV (n = 54)	Participants Without HCV (n = 54)	*P*-Value
**Liver fat fraction (%)**	12.4 (9.3, 18.0)	10.9 (8.7, 13.3)	0.39
Range	6.4-31.0	5.8-32.7	--
Missing (n, %)	0 (0%)	1 (1.9%)	0.32
**Liver volume (cc)**	1015.1 (884, 1283)	1086.9 (976, 1336)	0.29
Missing (n, %)	0 (0%)	1 (1.9%)	0.32
**SubQ fat cross-sectional area (cm**^**2**^)	288.4 (192.9, 435.3)	364.4 (208.3, 528.2)	0.14
Missing (n, %)	2 (3.7%)	1 (1.9%)	0.56
**SubQ fat area/total body area (%)**	43.4 (34.9, 48.9)	48.1 (37.7, 56.9)	0.28
Missing (n, %)	2 (3.7%)	1 (1.9%)	0.56
**Visceral fat cross-sectional area (cm**^**2**^)	101.9 (71.5, 145.3)	112.9 (70.1, 176.8)	0.50
Missing (n, %)	2 (3.7%)	1 (1.9%)	0.56
**Visceral fat area/total body area (%)**	14.3 (10.5, 19.2)	15.5 (9.9, 20.4)	0.50
Missing (n, %)	2 (3.7%)	1 (1.9%)	0.56

Abbreviations: HCV=hepatitis c virus; SubQ=subcutaneous

^a^Results reported as median (IQR) unless otherwise noted.

**Table 3:** Unadjusted and adjusted mean difference (95% confidence interval) in liver fat fraction between participants with and without chronic hepatitis C virus infection. Each model is adjusted for the variables indicated.

Characteristics	Mean Difference (95% CI) in Liver Fat Fraction (%)					
	Model #1		Model #2^a^		Model #3	
	Unadjusted Estimate (R^2^ = 0.020)	*P-Value*	Adjusted Estimate (R^2^ = 0.476)	*P-Value*	Adjusted Estimate (R^2^ = 0.261)	*P-Value*
Chronic hepatitis C virus infection	1.70 (−0.58, 3.98)	0.142	2.28 (0.55, 4.02)	0.011	2.77 (0.62, 4.93)	0.012
Male sex	--	--	2.23 (0.30, 4.16)	0.024	−1.03 (−3.26, 1.20)	0.362
Age (per 10 years)	--	--	0.61 (0.03, 1.20)	0.041	0.06 (−0.72, 0.83)	0.884
Body mass index (continuous; kg/m^2^)	--	--	0.67 (0.52, 0.82)	<0.001	--	--
Visceral fat cross-sectional area (per 10 cm^2^)	--	--	--	--	0.36 (0.22, 0.50)	<0.001

Abbreviations: CI = confidence interval

^a^Primary model

In our exploratory analysis stratifying the HCV group by HIV status, there was no difference in liver fat fraction between participants with HCV/HIV coinfection (n = 12) and participants without HCV (n = 54; mean difference = 0.51% [95% CI: −2.31, 3.34%]). Moreover, there was no difference in liver fat fraction between participants with HCV/HIV coinfection and participants with HCV monoinfection (n = 42; mean difference = −2.26% [95% CI: −5.11, 0.60%]). When we stratified the HCV group by liver fibrosis stage, the mean liver fat fraction was 5.34% (95% CI: 1.83, 8.84) higher for participants with chronic HCV and advanced hepatic fibrosis/cirrhosis, and 1.71% (95% CI: −0.51, 3.92) higher for participants with chronic HCV and no/minimal hepatic fibrosis, compared to participants without HCV, after adjustment for age, sex, and visceral fat cross-sectional area (S1 Table). Among participants with chronic HCV, there was no association between log HCV RNA and liver fat fraction (S2 Table).

### Mean differences in log cytokines and log IGF-1 Levels, by HCV status

Compared to participants without HCV, mean log IL-18 and TNF-α levels were significantly higher and mean log IGF-1 levels were significantly lower among participants with chronic HCV (Table 4). After adjustment for age, sex, and BMI, mean log levels of IL-18 and TNF-α remained significantly higher and mean log levels of IGF-1 remained significantly lower in participants with chronic HCV (Table 5). No statistically significant differences in mean log levels of IL-6 were observed between participants with HCV infection and those without HCV.

**Table 4:** Median and mean log values of cytokines and insulin-like growth factor-1, by chronic hepatitis C virus infection status.

Laboratory Assay^a^	Participants with Chronic HCV (n = 54)	Participants without HCV (n = 54)	*P-*Value
**Interleukin-6** (pg/mL)			
Median (IQR)	2.5 (1.3, 4.8)	2.5 (1.4, 4.2)	--
Mean log (95% CI)	0.46 (0.34, 0.59)	0.35 (0.25, 0.45)	0.15
**Interleukin-18** (pg/mL)			
Median (IQR)	241.0 (198.0, 391.0)	180.0 (137.0, 230.0)	--
Mean log (95% CI)	2.41 (2.34, 2.49)	2.27 (2.21, 2.32)	0.002
**Tumor necrosis factor-α** (pg/mL)			
Median (IQR)	12.6 (9.6, 14.2)	9.2 (7.3, 10.8)	--
Mean log (95% CI)	1.07 (1.03, 1.11)	0.96 (0.93, 0.99)	<.0001
**Insulin-like growth factor-1** (ng/mL)			
Median (IQR)	91.8 (62.9, 118.7)	132.5 (96.6, 183.3)	--
Mean log (95% CI)	1.93 (1.86, 2.00)	2.12 (2.07, 2.18)	<.0001
**Missing** (n, %)	9 (16.7%)	1 (1.9%)	0.008

Abbreviations: CI = confidence interval; HCV = hepatitis C virus; IQR = interquartile range

^a^Laboratory assay ranges: interleukin-6, 0.28–2,652 pg/mL; interleukin-18, 0.96–3,660 pg/mL; tumor necrosis factor-α, 0.3–1,160 pg/mL; insulin-like growth factor, 7–1,600 ng/mL

**Table 5:** Unadjusted and adjusted mean differences in log levels of cytokines and insulin-like growth factor-1 between participants with and without chronic hepatitis C virus infection.

Model	Log Interleukin-6		Log Interleukin-18		Log TNF-α		Log IGF-1	
	Mean Difference (95% CI)	*P*-Value	Mean Difference (95% CI)	*P*-Value	Mean Difference (95% CI)	*P*-Value	Mean Difference (95% CI)	*P*-Value
HCV, unadjusted	0.11 (−0.04, 0.27)	0.15	0.15 (0.06, 0.23)	0.002	0.11 (0.06, 0.16)	<.0001	−0.19 (−0.28, −0.11)	<.0001
HCV, adjusted for age and sex	0.12 (−0.03, 0.27)	0.12	0.14 (0.05, 0.24)	0.003	0.11 (0.06, 0.16)	<.0001	−0.18 (−0.26, −0.11)	<.0001
HCV, adjusted for age, sex, and body mass index^a^	0.13 (−0.01, 0.27)	0.07	0.14 (0.05, 0.24)	0.003	0.11 (0.06, 0.16)	<.0001	−0.18 (−0.26, −0.11)	<.0001

Abbreviations: CI = confidence interval; HCV = hepatitis C virus; IGF-1 = insulin-like growth factor-1; TNF-α = tumor necrosis factor-α

^a^Primary model

Among participants with chronic HCV, mean log IL-6 was higher in magnitude among those with advanced hepatic fibrosis/cirrhosis, while mean log IGF-1 was lower in magnitude, compared to participants with chronic HCV who had no/minimal hepatic fibrosis (Table 6).

**Table 6:** Median and mean log levels of cytokines and insulin-like growth factor-1 according to liver fibrosis stage among those with chronic hepatitis C virus infection.

Laboratory Assay	No or Minimal Hepatic Fibrosis (METAVIR Stage F0-F2) (n = 42)			Advanced Hepatic Fibrosis/Cirrhosis (METAVIR Stage F3-F4) (n = 11)		
	n^a^	Median (IQR)	Mean Log (95% CI)	n^a^	Median (IQR)	Mean Log (95% CI)
Interleukin-6	35	2.1 (1.2-4.2)	0.40 (0.26, 0.55)	10	4.7 (2.5-8.3)	0.69 (0.44, 0.93)
Interleukin-18	35	241 (198-405)	2.41 (2.34, 2.49)	10	256 (163-391)	2.41 (2.19, 2.63)
Tumor necrosis factor-α	34^b^	11.4 (9.53-14.2)	1.06 (1.01, 1.11)	10	13.1 (11.4-14.2)	1.09 (1.02, 1.16)
Insulin-like growth factor-1	35	96.7 (72.4-128.6)	1.99 (1.92, 2.06)	10	51.2 (33.8-90.2)	1.73 (1.56, 1.90)

Abbreviations CI=confidence interval; IQR=interquartile range

^a^Nine participants are not included as they did not have their blood drawn.

^b^One participant’s level of TNF-α was an outlier and was excluded.

### Associations between liver fat fraction and log cytokines and log IGF-1

After adjustment for age, sex, BMI, and HCV status, higher log IL-6, IL-18, and TNF-α levels were not associated with higher liver fat fraction (S3 Table). Similarly, there was no association between log IGF-1 level and liver fat fraction after adjusting for age, sex, BMI, and HCV status.

### Mean differences in subcutaneous and visceral adipose tissue, by HCV Status

Abdominal subcutaneous adipose tissue and visceral adipose tissue cross-sectional area by MRI did not differ by chronic HCV status (Table 2). Chronic HCV was associated with lower visceral adipose tissue cross-sectional area, but not abdominal subcutaneous adipose tissue cross-sectional area, after adjustment for age, sex, and BMI (S4 Table). The association between chronic HCV and lower visceral adipose tissue cross-sectional area was stronger when limiting to chronic HCV participants with advanced hepatic fibrosis/cirrhosis and comparing to controls.

## Discussion

In this cross-sectional study, we found that significant hepatic steatosis, determined by liver fat fraction via MRI using a three-dimensional volumetric evaluation, was highly prevalent in both participants with untreated chronic HCV and those without HCV infection. After controlling for age, sex, and BMI, we found that chronic HCV was associated with higher (2.28%) liver fat fraction. This association remained in sensitivity analyses replacing BMI with visceral fat cross-sectional area. While this observation was statistically significant, this difference in liver fat fraction is unlikely to be clinically relevant. Chronic HCV was associated with higher mean levels of the inflammatory cytokines IL-18 and TNF-α, and lower mean levels of IGF-1. However, there were no associations between liver fat fraction and either the levels of any of these inflammatory cytokines or IGF-1.

Few studies have evaluated the quantitative difference in hepatic fat content between patients with and without chronic HCV [9,38] and, to our knowledge, none have employed a three-dimensional volumetric method using MRI to evaluate comprehensively the differences in liver fat fraction between these groups. Unlike magnetic resonance spectroscopy or liver biopsy, which quantify fat in limited regions of the liver, this three-dimensional volumetric process using MRI allows for assessment of fat throughout the entire liver [39–41]. We found that chronic HCV was associated with a 2.28% higher mean liver fat fraction, an association observed despite similar prevalence of metabolic comorbidities among controls. This finding contrasts with prior work employing two-dimensional MRI among participants with and without HCV [9]. Among 57 participants with HCV, liver fat fraction was 28% lower in participants with HCV monoinfection when compared to 107 participants without HCV or HIV. The reason for this difference is likely due to the higher prevalence of metabolic comorbidities in our participants. A prior study of participants undergoing transient elastography with controlled attenuation parameter (CAP) to quantify hepatic steatosis found that the median CAP score was 21.9 dB/m higher among people with HCV compared to people without HCV despite both groups having similar age, sex, and BMI [38]. Taken together, these findings suggest that viral-associated pathologic mechanisms may modestly contribute to the development of HCV-associated steatosis [8].

Our three-dimensional volumetric evaluation approach for liver fat fraction identified significant hepatic steatosis at the 5% threshold in all participants with and without HCV infection. While the severity of steatosis in both groups did not exceed a liver fat fraction of 33%, our finding highlights the prevalence of hepatic steatosis and suggests that it will continue to be an important contributor to health outcomes in the future. Steatotic liver disease is associated with systemic inflammation [42], cardiovascular disease [43], and end-stage liver disease [44,45]; however, the full spectrum of impact of hepatic steatosis on health outcomes warrants further examination.

In addition to its association with hepatic inflammation, we hypothesized that chronic HCV would also be associated with systemic inflammation, specifically with elevated levels of inflammatory cytokines. We evaluated IL-6, IL-18, and TNF-a because these might promote intra-hepatic lipid peroxidation and peripheral lipolysis, which could contribute to development of hepatic steatosis [15]. Our study found that participants with chronic HCV had higher levels of IL-18 and TNF-α, but not IL-6, compared to participants without HIV or HCV. However, no associations were observed between levels of any of the inflammatory cytokines and liver fat fraction. Our findings suggest that systemic measures of IL-6, IL-18, TNF-α are not associated with greater hepatic steatosis. Further work is needed to characterize inflammatory pathways and evaluate other mechanisms of hepatic fat deposition in people with chronic HCV.

The liver is a primary source of IGF-1 and contributes to metabolic regulation [16]. We hypothesized that chronic HCV infection, through persistent hepatic inflammation and fibrosis, would be associated with decreased production of this growth factor, which might lead to impaired liver fat regulation and promote hepatic steatosis [46]. Consistent with our hypothesis, we observed that chronic HCV was associated with lower IGF-1 levels, with the strongest association observed among participants with advanced hepatic fibrosis/cirrhosis. However, we observed no association between IGF-1 and liver fat fraction. While prior work has also implicated inflammasome activation in the development or hepatic steatosis [47], our findings do not suggest systemic measures of IL-6, IL-18, TNF-α are associated with greater hepatic fat deposition.

Our study has several potential limitations. First, the cross-sectional design did not allow us to evaluate changes in three-dimensional volumetric liver fat fraction by MRI and other hepatic steatosis risk factors over time. Second, the small sample size may have limited the power of our analyses to detect differences. Third, our analyses did not account for duration of chronic HCV, since the date of HCV infection is challenging to ascertain, socioeconomic status, food security, or poor nutrition, which are factors that might be important contributors to liver fat fraction. Finally, our study was overly representative of participants who were middle-aged, Black or African American, with overweight BMI, which may limit generalizability beyond these groups. However, the predominance of recent HCV diagnoses in our study participants likely reflect people with HCV diagnosed through universal HCV screening and novel screening programs [48,49]. Moreover, the majority of participants had limited hepatic fibrosis, reflecting the improved access to direct-acting antiviral therapy [50–52]. Both of these themes are representative of patients currently accessing HCV care in the United States. While our study is also not generalizable to patients with chronic HCV who received previous treatment with direct-acting antiviral therapy, future studies should examine three-dimensional volumetric measurements using MRI in this group.

Our study had a number of strengths. We performed a comprehensive evaluation of body fat depots among participants with and without chronic HCV, which allowed a more careful examination of the magnitude of differences in liver fat fraction and other fat depots between these groups. We used MRI to measure three-dimensional volumetric liver fat fraction, a novel approach for assessment of fat throughout the hepatic parenchyma. Our approach employed manual shimming and transmitter adjustments prior to each acquisition, which improved the stability of the reconstruction algorithm, and we used the lowest flip angle that provided sufficient signal-to-noise ratio for imaging [40,41,53,54]. Moreover, our analyses accounted for important confounding variables, including BMI, and visceral fat cross-sectional area by MRI.

## Conclusion

We found that treatment-naïve chronic HCV infection was associated with a higher mean volumetric liver fat fraction as determined by MRI, though this difference is unlikely to be clinically significant. Participants with chronic HCV had higher mean levels of IL-18 and TNF-α, reflective of proinflammatory cascades potentiated by the virus, and lower mean levels of IGF-1, likely due to decreased hepatic synthesis in the setting of HCV-associated inflammation and fibrosis. Further research is needed to identify alternative mechanisms that potentiate liver fat deposition with chronic HCV infection.

## Supporting information

S1 Fig Example Liver Magnetic Resonance Images and Parameter Maps for Participants with and without HCV. On the left, images are displayed for a 24-year-old male participant without HCV and a BMI of 25 kg/m^2^. On the right, images are displayed for a 30-year-old male participant with HCV and a BMI of 21 kg/m^2^. The top two rows display the out-of-phase echo at 3.9 milliseconds and the in-phase echo at 4.6 milliseconds. The bottom row displays the proton-density fat fraction percentage maps with the manually drawn liver boundary superimposed in white.
(TIF)

S2 Fig Patient Recruitment Flow. Abbreviations: HCV = hepatitis C virus; HIV = human immunodeficiency virus; RNA, ribonucleic acid. *See S1 File for a list of participant screening questions.
(TIF)

S3 Fig Compartment Analysis of Abdominal Subcutaneous Cross-Sectional Area and Visceral Fat Cross-Sectional Area by MRI. Panel A shows representative abdominal subcutaneous compartment (blue) and visceral compartment (red) for participants without HCV and with HCV. Panel B displays the calculation for total abdominal cross-sectional area (green), measured in cross-sectional area (cm^2^). For compartment analysis, a single magnetic resonance imaging slice at the level of umbilicus was used to define cross-sectional areas.
(TIF)

S1 Table 1. Adjusted mean difference (95% confidence interval) in liver fat fraction between participants without hepatitis C virus infection and participants with: 1) chronic hepatitis C virus infection with no/minimal hepatic fibrosis, and 2) chronic hepatitis C virus infection with advanced hepatic fibrosis/cirrhosis. Each model is adjusted for the variables indicated.
(DOCX)

S2 Table 2. Association between log hepatitis C virus (HCV) RNA and liver fat fraction among participants with chronic HCV infection. (DOCX)

S3 Table Adjusted mean difference (95% confidence interval) in liver fat fraction per log level of specified cytokine and insulin-like growth factor-1. Each model is adjusted for the variables indicated.
(DOCX)

S4 Table Adjusted mean differences in abdominal subcutaneous and visceral adipose tissue cross-sectional area between participants with and without chronic hepatitis C virus infection. (DOCX)

S1 File Participant Screening Questions. (DOCX)

S2 File Supplementary MRI Methods. (DOCX)
