# Supplementary figures and images for: Magnetic resonance evaluation of three-dimensional liver fat fraction by hepatitis C status and associations with inflammatory cytokines
Source: PLoS One. 2025 Jul 23;20(7):e0327668. doi: 10.1371/journal.pone.0327668 (PMC12286359; doi:10.1371/journal.pone.0327668)

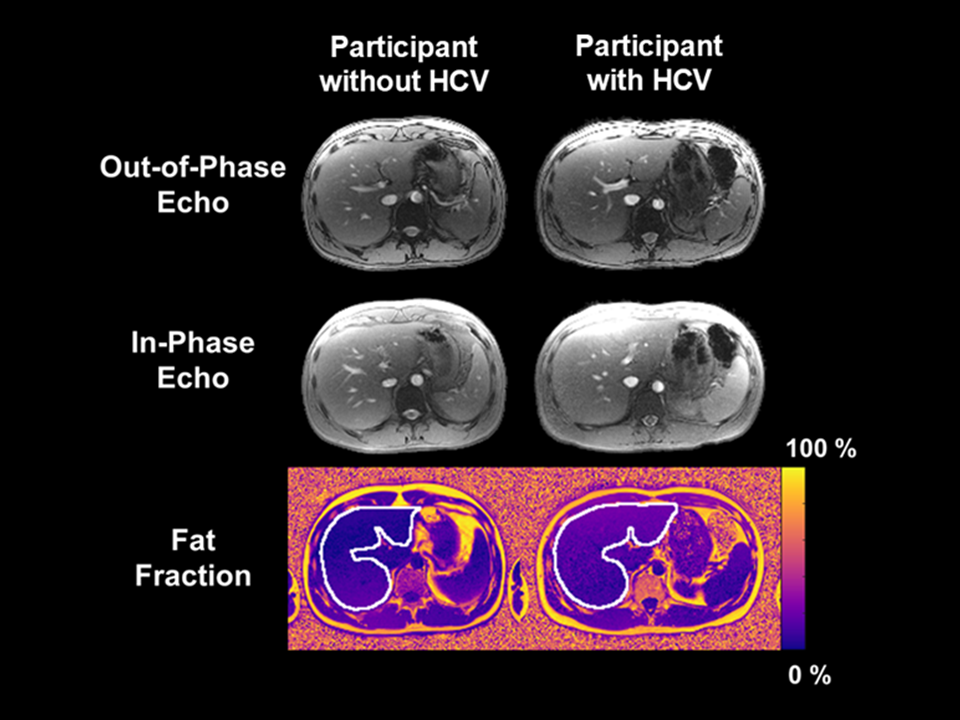

Supplement: S1 Fig — On the left, images are displayed for a 24-year-old male participant without HCV and a BMI of 25 kg/m2. On the right, images are displayed for a 30-year-old male participant with HCV and a BMI of 21 kg/m2. The top two rows display the out-of-phase echo at 3.9 milliseconds and the in-phase echo at 4.6 milliseconds. The bottom row displays the proton-density fat fraction percentage maps with the manually drawn liver boundary superimposed in white. (TIF) [file pone.0327668.s001.tif]

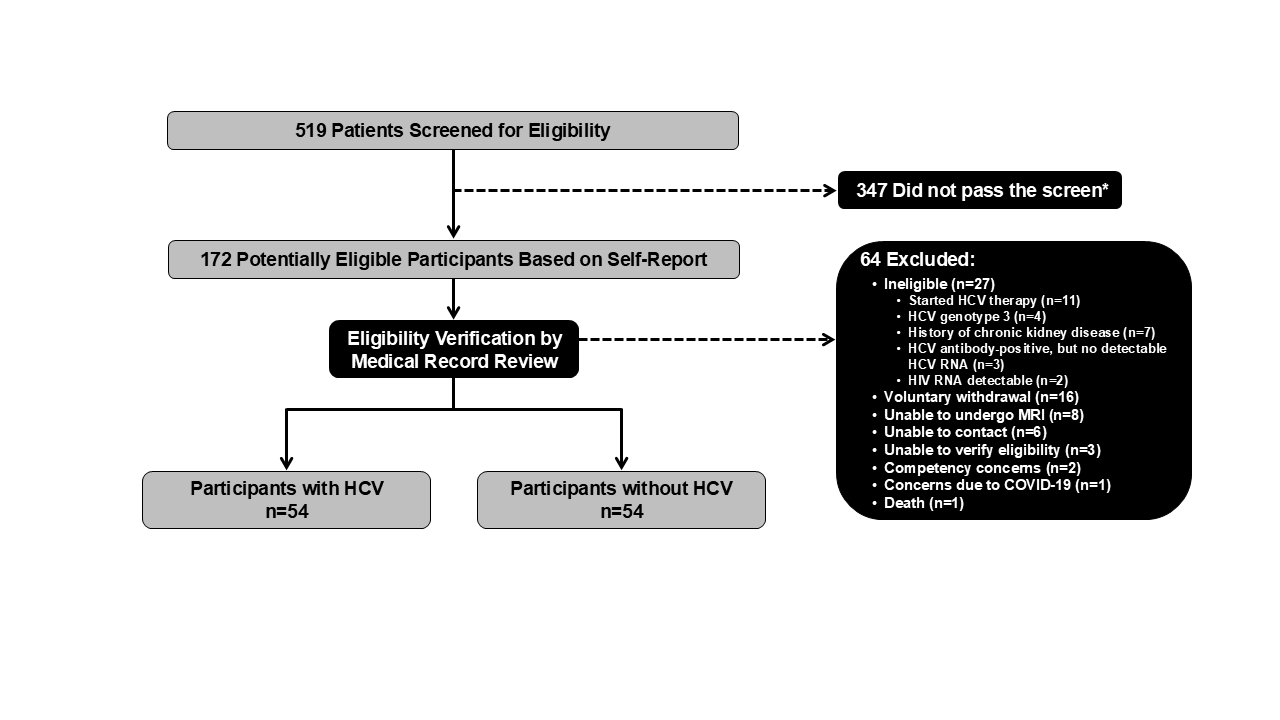

Supplement: S2 Fig — Abbreviations: HCV = hepatitis C virus; HIV = human immunodeficiency virus; RNA, ribonucleic acid. *See S1 File for a list of participant screening questions. (TIF) [file pone.0327668.s002.tif]

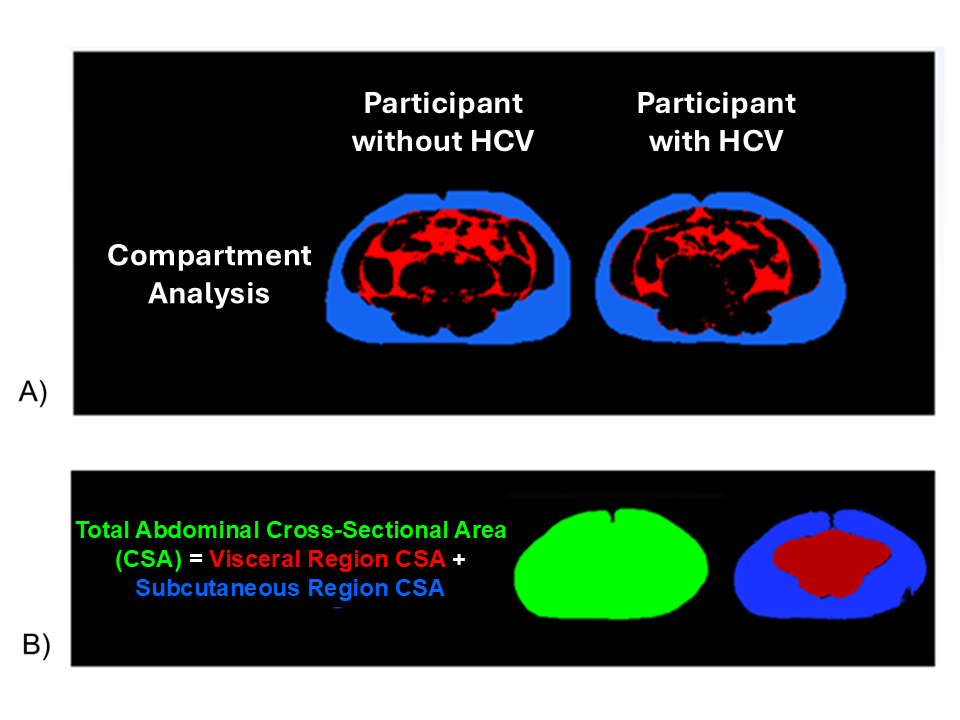

Supplement: S3 Fig — Panel A shows representative abdominal subcutaneous compartment (blue) and visceral compartment (red) for participants without HCV and with HCV. Panel B displays the calculation for total abdominal cross-sectional area (green), measured in cross-sectional area (cm2). For compartment analysis, a single magnetic resonance imaging slice at the level of umbilicus was used to define cross-sectional areas. (TIF) [file pone.0327668.s003.tif]
