# Supplementary material for: Magnetic resonance evaluation of three-dimensional liver fat fraction by hepatitis C status and associations with inflammatory cytokines
Source: PLoS One. 2025 Jul 23;20(7):e0327668. doi: 10.1371/journal.pone.0327668 (PMC12286359; doi:10.1371/journal.pone.0327668)
Supplement: S1 Table — Each model is adjusted for the variables indicated. (DOCX) [file pone.0327668.s004.docx]

**Magnetic Resonance Evaluation of Three-Dimensional Liver Fat Fraction by Hepatitis C Status and Associations with Inflammatory Cytokines**

Jessie Torgersen, MD, MHS, MSCE; Craig W. Newcomb, MS; Dean M. Carbonari, MS; Shanae M. Smith, MHA; Katherine L. Brecker, BS; Chamith S. Rajapakse, PhD; Brandon C. Jones; Christiana Cottrell; Rasleen Grewal; Jennifer C. Price, MD, PhD; Joshua F. Baker, MD, MSCE; Jay R. Kostman, MD; Stacey Trooskin, MD, PhD; Rebecca A. Hubbard, PhD; Babette S. Zemel, PhD; Mary B. Leonard, MD, MSCE; Vincent Lo Re III, MD, MSCE

# **Supplementary Table 1. Adjusted mean difference (95% confidence interval) in liver fat fraction between participants without hepatitis C virus infection and participants with: 1) chronic hepatitis C virus infection with no/minimal hepatic fibrosis, and 2) chronic hepatitis C virus infection with advanced hepatic fibrosis/cirrhosis.** Each model is adjusted for the variables indicated.

|  | **Mean Difference (95% CI) in Liver Fat Fraction (%)** | | | | | |
| --- | --- | --- | --- | --- | --- | --- |
| **Variables** | **Model #1** | | **Model #2^a^** | | **Model #3** | |
|  | **Unadjusted Estimate** | ***P*** | **Adjusted**  **Estimate** | ***P*** | **Adjusted**  **Estimate** | ***P*** |
| Chronic hepatitis C virus infection |  | 0.041 |  | 0.055 |  | 0.010 |
| No/minimal hepatic fibrosis | 0.50 (-1.80, 2.81) |  | 1.95 (0.13, 3.76) |  | 1.71 (-0.51, 3.92) |  |
| Advanced hepatic fibrosis/cirrhosis | 4.76 (1.06, 8.46) |  | 2.47 (-0.47, 5.40) |  | 5.34 (1.83, 8.84) |  |
| Male sex | -- | -- | 2.23 (0.30, 4.16) | 0.024 | -1.03 (-3.26, 1.20) | 0.362 |
| Age (per 10 years) | -- | -- | 0.61 (0.03, 1.20) | 0.041 | 0.06 (-0.72, 0.83) | 0.884 |
| Body mass index (continuous; kg/m^2^) | -- | -- | 0.67 (0.52, 0.82) | <0.001 | -- | -- |
| Visceral fat cross-sectional area (per 10 cm^2^) | -- | -- | -- | -- | 0.36 (0.22, 0.50) | <0.001 |

Abbreviations: CI=confidence interval

^a^ Primary model
