# Supplementary material for: Magnetic resonance evaluation of three-dimensional liver fat fraction by hepatitis C status and associations with inflammatory cytokines
Source: PLoS One. 2025 Jul 23;20(7):e0327668. doi: 10.1371/journal.pone.0327668 (PMC12286359; doi:10.1371/journal.pone.0327668)
Supplement: S2 Table — (DOCX) [file pone.0327668.s005.docx]

**Magnetic Resonance Evaluation of Three-Dimensional Liver Fat Fraction by Hepatitis C Status and Associations with Inflammatory Cytokines**

Jessie Torgersen, MD, MHS, MSCE; Craig W. Newcomb, MS; Dean M. Carbonari, MS; Shanae M. Smith, MHA; Katherine L. Brecker, BS; Chamith S. Rajapakse, PhD; Brandon C. Jones; Christiana Cottrell; Rasleen Grewal; Jennifer C. Price, MD, PhD; Joshua F. Baker, MD, MSCE; Jay R. Kostman, MD; Stacey Trooskin, MD, PhD; Rebecca A. Hubbard, PhD; Babette S. Zemel, PhD; Mary B. Leonard, MD, MSCE; Vincent Lo Re III, MD, MSCE

#

# **Supplementary Table 2. Association between log hepatitis C virus (HCV) RNA and liver fat fraction among participants with chronic HCV infection.**

| **Model** | **Mean difference (95% CI)** | ***P*-Value** |
| --- | --- | --- |
| Log HCV RNA IU/mL, unadjusted | 0.18 (-0.78, 1.14) | 0.71 |
| Log HCV RNA IU/mL, adjusted for sex, age (continuous) | -0.09 (-1.11, 0.92) | 0.86 |
| Log HCV RNA IU/mL, adjusted for sex, age (continuous), body mass index (continuous) | -0.08 (-0.77, 0.61) | 0.82 |

Abbreviations: CI=confidence interval; HCV=hepatitis C virus; IU=international units; RNA=ribonucleic acid
