# Supplementary material for: Magnetic resonance evaluation of three-dimensional liver fat fraction by hepatitis C status and associations with inflammatory cytokines
Source: PLoS One. 2025 Jul 23;20(7):e0327668. doi: 10.1371/journal.pone.0327668 (PMC12286359; doi:10.1371/journal.pone.0327668)
Supplement: S3 Table — Each model is adjusted for the variables indicated. (DOCX) [file pone.0327668.s006.docx]

**Magnetic Resonance Evaluation of Three-Dimensional Liver Fat Fraction by Hepatitis C Status and Associations with Inflammatory Cytokines**

Jessie Torgersen, MD, MHS, MSCE; Craig W. Newcomb, MS; Dean M. Carbonari, MS; Shanae M. Smith, MHA; Katherine L. Brecker, BS; Chamith S. Rajapakse, PhD; Brandon C. Jones; Christiana Cottrell; Rasleen Grewal; Jennifer C. Price, MD, PhD; Joshua F. Baker, MD, MSCE; Jay R. Kostman, MD; Stacey Trooskin, MD, PhD; Rebecca A. Hubbard, PhD; Babette S. Zemel, PhD; Mary B. Leonard, MD, MSCE; Vincent Lo Re III, MD, MSCE

#

# **Supplementary Table 3. Adjusted mean difference (95% confidence interval) in liver fat fraction per log level of specified cytokine and insulin-like growth factor-1. Each model is adjusted for the variables indicated.**

|  | **Unadjusted** | | **Age-/Sex-Adjusted** | | **Age-/Sex-/BMI-Adjusted** | | **Age-/Sex-/BMI-/HCV-Adjusted^*^** | |
| --- | --- | --- | --- | --- | --- | --- | --- | --- |
| **Laboratory Assay** | **Mean Difference (95% CI)** | ***P*-Value** | **Mean Difference (95% CI)** | ***P*-Value** | **Mean Difference (95% CI)** | ***P*-Value** | **Mean Difference (95% CI)** | ***P*-Value** |
| Interleukin-6 (pg/mL) | 6.28 (3.40, 9.16) | <.0001 | 5.80 (2.63, 8.98) | 0.001 | 2.35 (-0.40, 5.09) | 0.09 | 1.76 (-0.97, 4.48) | 0.20 |
| Interleukin-18 (pg/mL) | 1.55 (-3.73, 6.83) | 0.56 | 1.66 (-3.57, 6.88) | 0.53 | 1.58 (-2.44, 5.60) | 0.44 | 0.10 (-3.97, 4.17) | 0.96 |
| Tumor necrosis factor-α (pg/mL) | 8.78 (-0.50, 18.1) | 0.06 | 8.33 (-0.85, 17.50) | 0.07 | 6.95 (-0.13, 14.03) | 0.05 | 3.59 (-4.08, 11.27) | 0.35 |
| Insulin-like growth factor-1 (ng/mL) | -6.32 (-11.2, -1.41) | 0.01 | -4.66 (-10.5, 1.23) | 0.12 | -3.90 (-8.43, 0.63) | 0.09 | -1.46 (-6.46, 3.54) | 0.56 |

Abbreviations: CI=confidence interval; IGF-1=insulin-like growth factor-1; TNF-α=tumor necrosis factor-α

^*^ Primary model
