# Supplementary material for: Magnetic resonance evaluation of three-dimensional liver fat fraction by hepatitis C status and associations with inflammatory cytokines
Source: PLoS One. 2025 Jul 23;20(7):e0327668. doi: 10.1371/journal.pone.0327668 (PMC12286359; doi:10.1371/journal.pone.0327668)
Supplement: S4 Table — (DOCX) [file pone.0327668.s007.docx]

**Magnetic Resonance Evaluation of Three-Dimensional Liver Fat Fraction by Hepatitis C Status and Associations with Inflammatory Cytokines**

Jessie Torgersen, MD, MHS, MSCE; Craig W. Newcomb, MS; Dean M. Carbonari, MS; Shanae M. Smith, MHA; Katherine L. Brecker, BS; Chamith S. Rajapakse, PhD; Brandon C. Jones; Christiana Cottrell; Rasleen Grewal; Jennifer C. Price, MD, PhD; Joshua F. Baker, MD, MSCE; Jay R. Kostman, MD; Stacey Trooskin, MD, PhD; Rebecca A. Hubbard, PhD; Babette S. Zemel, PhD; Mary B. Leonard, MD, MSCE; Vincent Lo Re III, MD, MSCE

#

# **Supplementary Table 4. Adjusted mean differences in abdominal subcutaneous and visceral adipose tissue cross-sectional area between participants with and without chronic hepatitis C virus infection.**

|  | **Subcutaneous Adipose Tissue**  **Cross-Sectional Area (cm^2^)** | | **Visceral Adipose Tissue**  **Cross-Sectional Area (cm^2^)** | |
| --- | --- | --- | --- | --- |
| **Model** | **Mean Difference (95% CI)** | ***P*-Value** | **Mean Difference (95% CI)** | ***P*-Value** |
| Chronic HCV, adjusted for age, sex, and BMI | -10.4 (-44.9, 24.19) | 0.5530 | -26.5 (-50.3, -2.71) | 0.0294 |
| Participants without HCV | Reference |  | Reference |  |
| Chronic HCV with low fibrosis, adjusted for age, sex, and BMI | -5.81 (-46.1, 34.49) | 0.9314 | -17.7 (-47.2, 11.82) | 0.0206 |
| Chronic HCV with high fibrosis, adjusted for age, sex, and BMI | 3.96 (-54.8, 62.72) |  | -61.5 (-105, -18.4) |  |
| Participants without HCV | Reference |  | Reference |  |

Abbreviations: BMI=body mass index; CI=confidence interval; HCV=hepatitis C virus
