# Supplementary material for: Magnetic resonance evaluation of three-dimensional liver fat fraction by hepatitis C status and associations with inflammatory cytokines
Source: PLoS One. 2025 Jul 23;20(7):e0327668. doi: 10.1371/journal.pone.0327668 (PMC12286359; doi:10.1371/journal.pone.0327668)
Supplement: S1 File — (DOCX) [file pone.0327668.s008.docx]

**Magnetic Resonance Evaluation of Three-Dimensional Liver Fat Fraction by Hepatitis C Status and Associations with Inflammatory Cytokines**

Jessie Torgersen, MD, MHS, MSCE; Craig W. Newcomb, MS; Dean M. Carbonari, MS; Shanae M. Smith, MHA; Katherine L. Brecker, BS; Chamith S. Rajapakse, PhD; Brandon C. Jones; Christiana Cottrell; Rasleen Grewal; Jennifer C. Price, MD, PhD; Joshua F. Baker, MD, MSCE; Jay R. Kostman, MD; Stacey Trooskin, MD, PhD; Rebecca A. Hubbard, PhD; Babette S. Zemel, PhD; Mary B. Leonard, MD, MSCE; Vincent Lo Re III, MD, MSCE

**Supplementary Methods: Participant Screening Questions**

1. Are you 18 years of age or older?

▪ No (Not Eligible)

▪ Yes

2. How much do you currently weigh (pounds)?

3. Are you currently pregnant or breastfeeding or plan to become pregnant over the next 18 months?

▪ No/Not Applicable

▪ Yes (Not Eligible)

4. Do you have any metal or metallic foreign objects in your body that you are unable to remove?

▪ No

▪ Yes (Not Eligible)

5. Are you able to undergo an MRI and do so without sedation?

▪ No (Not Eligible)

▪ Yes

6. Have you ever had or been diagnosed with chronic kidney disease?

▪ No

▪ Yes (Not Eligible)

7. Have you ever had or been diagnosed with hepatitis B virus infection (HBV)?

▪ No

▪ Yes (Not Eligible)

8. Have you ever had or been diagnosed with celiac disease?

▪ No

▪ Yes (Not Eligible)

9. Have you ever had or been diagnosed with small bowel resection surgery?

▪ No

▪ Yes (Not Eligible)

10. Have you ever had or been diagnosed with chronic diarrhea?

▪ No

▪ Yes (Not Eligible)

11. Have you had weight loss greater than 5% of your body weight within the past 3 months?

▪ No

▪ Yes (Not Eligible)

12. Have you ever had or been diagnosed with cancer?

▪ No

▪ Yes

a) What type or types of cancer have you ever been diagnosed with?

○ Non-melanoma skin cancer ONLY

○ All other cancers (Not Eligible)

13. Have you ever been diagnosed with HIV?

▪ No

▪ Yes

a) Are you currently on an antiretroviral (ART) regimen?

○ No (Not Eligible)

○ Yes

b) Is your HIV viral load undetectable?

○ No (Not Eligible)

○ Yes

14. Have you ever been diagnosed with hepatitis C (HCV)?

▪ No

▪ Yes

a) Have you ever received treatment for your HCV with oral direct acting antiviral

(DAA) therapy?

○ No

○ Yes (Not Eligible)

b) Does your provider plan on treating your HCV with oral direct acting antiviral

(DAA) therapy?

○ No (Not Eligible)

○ Yes

○ Don't Know
