# Supplementary material for: Magnetic resonance evaluation of three-dimensional liver fat fraction by hepatitis C status and associations with inflammatory cytokines
Source: PLoS One. 2025 Jul 23;20(7):e0327668. doi: 10.1371/journal.pone.0327668 (PMC12286359; doi:10.1371/journal.pone.0327668)
Supplement: S2 File — (DOCX) [file pone.0327668.s009.docx]

**Magnetic Resonance Evaluation of Three-Dimensional Liver Fat Fraction by Hepatitis C Status and Associations with Inflammatory Cytokines**

Jessie Torgersen, MD, MHS, MSCE; Craig W. Newcomb, MS; Dean M. Carbonari, MS; Shanae M. Smith, MHA; Katherine L. Brecker, BS; Chamith S. Rajapakse, PhD; Brandon C. Jones; Christiana Cottrell; Rasleen Grewal; Jennifer C. Price, MD, PhD; Joshua F. Baker, MD, MSCE; Jay R. Kostman, MD; Stacey Trooskin, MD, PhD; Rebecca A. Hubbard, PhD; Babette S. Zemel, PhD; Mary B. Leonard, MD, MSCE; Vincent Lo Re III, MD, MSCE

**Supplementary Methods: MRI Measurement of Hepatic and Visceral Adipose Depots**

All MRI scans were performed in the head-first supine position with a spine coil and an 18-element flexible body array placed anterior to the abdomen. Manual shimming and transmitter adjustments were performed before each scan to improve B0 magnetic field homogeneity and signal-to-noise ratio. Axial multi-echo gradient-echo sequences in the liver and abdomen were acquired under free breathing with the following sequence parameters: repetition time 7.1 milliseconds, field-of-view 487x325 mm, matrix 240x160, bandwidth 1360 Hz/Pixel, flip angle 12 degrees, nominal voxel size 2 mm in-plane, 5 mm slice thickness. Two separate 3-echo sequences were run for each acquisition to allow for interleaving echo times of 1.8, 3.2, 4.6 milliseconds, and 2.5, 3.9, 5.3 milliseconds. Liver scans consisted of a variable number of multi-slice acquisitions to cover the entire liver volume, with a minimum of 35 contiguous slices for the smallest livers. An open-sourced offline reconstruction toolbox based on the Iterative Decomposition of water and fat with Echo Asymmetry and Least-squares (IDEAL) algorithm was used to reconstruct the k-space into parametric fat fraction maps.[1-5] Briefly, the IDEAL method uses the multi-channel, multi-echo complex data to simultaneously fit the fat fraction and T_2_* signal decay within a voxel by assuming the multipeak fat model is known *a priori*. This generated proton-density fat fraction (PDFF%) parametric maps, where 0% corresponds to voxels comprised entirely of water and 100% corresponds to voxels entirely comprised of fat.

Abdomen scans consisted of a single two-dimensional slice acquired at the level of the umbilicus, from which VAT and SAT were measured as percentages of total body cross sectional area. For fat fraction parameter maps, we implemented a threshold of 50% (i.e., selecting voxels containing more fat signal than water signal) to reliably separate voxels containing fat tissue from those containing normal soft tissue. Radiology research technicians manually segmented the boundary between the visceral and subcutaneous compartments from the fourth echo time image, which is the echo where fat and water were most out-of-phase.[6] Additionally, fatty voxels within the spine and small regions with air-tissue susceptibility speckling artifacts were manually segmented and removed to generate clean fat maps without errors. Final segmentations were visually verified for accuracy by a radiology researcher with over two decades of experience in body MRI research (**Supplementary Figure 3**).

**REFERENCES**

1. Reeder, S.B., et al., *Multicoil Dixon chemical species separation with an iterative least-squares estimation method.* Magn Reson Med, 2004. **51**(1): p. 35-45.

2. Yu, H., et al., *Multiecho reconstruction for simultaneous water-fat decomposition and T2* estimation.* J Magn Reson Imaging, 2007. **26**(4): p. 1153-61.

3. Yu, H., et al., *Multiecho water-fat separation and simultaneous R2* estimation with multifrequency fat spectrum modeling.* Magn Reson Med, 2008. **60**(5): p. 1122-34.

4. Hu, H.H., et al., *ISMRM workshop on fat-water separation: insights, applications and progress in MRI.* Magn Reson Med, 2012. **68**(2): p. 378-88.

5. Hernando, D., et al., *Robust water/fat separation in the presence of large field inhomogeneities using a graph cut algorithm.* Magn Reson Med, 2010. **63**(1): p. 79-90.

6. Boehm, C., et al., *On the water–fat in-phase assumption for quantitative susceptibility mapping.* Magnetic Resonance in Medicine, 2023. **89**(3): p. 1068-1082.
